# Content and Themes of Repetitive Thinking in Postnatal First-Time Mothers

**DOI:** 10.3389/fpsyg.2021.586538

**Published:** 2021-02-02

**Authors:** Jill M. Newby, Aliza Werner-Seidler, Melissa J. Black, Colette R. Hirsch, Michelle L. Moulds

**Affiliations:** ^1^Black Dog Institute, The University of New South Wales, Sydney, NSW, Australia; ^2^Institute of Psychiatry, Psychology and Neuroscience, King’s College London, London, United Kingdom; ^3^School of Psychology, The University of New South Wales, Sydney, NSW, Australia

**Keywords:** repetitive thinking, perinatal, depression, anxiety, rumination

## Abstract

Repetitive thinking (RT) predicts and maintains depression and anxiety, yet the role of RT in the perinatal context has been under-researched. Further, the content and themes that emerge during RT in the perinatal period have been minimally investigated. We recruited an online community sample of women who had their first baby within the past 12 months (*n* = 236). Participants completed a battery of self-report questionnaires which included four open-ended questions about the content of their RT. Responses to the latter were analyzed using an inductive thematic analysis approach. Participants reported RT about a range of unexpected emotional responses to becoming a new mother, impact on their sleep and cognitive functioning, as well as the impact on their identity, sense of self, lifestyle, achievements, and ability to function. RT was commonly experienced in first-time mothers, and the themes that emerged conveyed an overall sense of discrepancy between expectations and reality, as well as adjustment to profound change. By providing insight into the content of RT in new mothers, the findings of our study have scope to inform the content of interventions that seek to prevent and treat postnatal mental health problems, particularly those which target key psychological processes such as RT.

## Introduction

Depressive rumination refers to the tendency to respond to sad mood and related symptoms by dwelling on their causes, meanings and consequences (Nolen-Hoeksema, 1991; Nolen-Hoeksema et al., 2008). Worry represents another example of repetitive thinking (RT) and is characterized by perseverative thinking about future threat. Both of these types of RT are well-established predictors of the onset and maintenance of depression and anxiety, respectively. To date, the nature, role and consequences of RT have been minimally studied in the context of perinatal psychological adjustment (see DeJong et al., 2016 for review; also, Moulds et al., 2018), although there is growing evidence that RT predicts depression in the perinatal period (DeJong et al., 2016). For example, worry in the first trimester predicts depression in the third trimester (Schmidt et al., 2016), and rumination predicts increases in depression symptoms from the third trimester to 8 weeks postpartum (an association that is not moderated by level of depression in the third trimester; Barnum et al., 2013). RT also interacts with other factors (e.g., level of social functioning; O’Mahen et al., 2010; perfectionism; Egan et al., 2017) to predict postnatal depression symptoms.

Repetitive thinking not only predicts postnatal psychological symptoms, it also maintains them. For example, RT impairs problem-solving ability and reduces confidence in problem-solving capacity (O’Mahen et al., 2015). Similarly, for women with postpartum generalized anxiety disorder (GAD), RT reduces responsivity to infants – suggesting a key role for RT in mother-infant bonding (Stein et al., 2012). Furthermore, RT has been hypothesized to play a key role in mediating the relationship between maternal psychopathology and parenting (Stein et al., 2009; Psychogiou and Parry, 2014), highlighting the potential importance of RT as a key cognitive process in the perinatal context.

In their review of the literature in this field, DeJong et al. (2016) highlighted several key gaps in current knowledge. One of these is the lack of research on the content and themes of RT in the context of postnatal depression. Although DeJong et al. (2016) cite qualitative work on women’s experience of PND that appears akin to ruminative themes (e.g., incongruity between the expectations and reality of pregnancy/motherhood: Beck, 2002), they draw attention to the fact that no research has directly examined the content of rumination in postpartum depression. In contrast, the content of repetitive thoughts has been investigated in the literature on obsessions and intrusive thoughts in perinatal samples (see Abramowitz et al., 2003, for a review). For example, in a treatment-seeking sample of postpartum women, Abramowitz et al. (2010) found that the majority experienced intrusive obsession-like thoughts (e.g., about Sudden Infant Death Syndrome [SIDS], the baby suffocating). Whilst we note studies in which investigators have examined the content of worry (one specific type of RT) in perinatal populations (e.g., Moran et al., 2014), as well as perinatal women with generalized anxiety disorder (GAD; Goldfinger et al., 2020), to our knowledge, no research to date has investigated the themes of RT (as defined more broadly here) in the context of postnatal anxiety. Indeed, the absence of such work reflects the relative neglect of anxiety in this literature (Moulds et al., 2018), despite the documented prevalence of anxiety across the perinatal period (e.g., Dennis et al., 2017; Fairbrother et al., 2017; Viswasam et al., 2019).

Accordingly, as a first step, there is a need to develop our understanding of the themes and experiences that are commonly the focus of RT in the postnatal period, in order to determine the types of thoughts that new mothers typically return to repeatedly. We therefore focus on an unselected sample of new mothers. Delineating the themes of RT that characterize such a sample is an important preliminary step before moving on to examine such themes in women with perinatal psychological disorders – an approach that we have taken in the context of depression (see Newby and Moulds, 2012). This preliminary work will provide an understanding of the typical nature of RT in postpartum and a foundation for developing effective interventions to target unhelpful RT, with the aim of preventing future psychological disorders in the perinatal period. Such future work will enable clinicians to be better placed to address unhelpful RT in new mothers.

The aim of our study was to delineate the themes and content of RT in a community-sample of new mothers with babies under 12 months old. We opted to administer the survey online to maximize the size of our sample, acknowledging the potential practical impediments to new mothers attending research sessions in the lab (e.g., time pressures, arranging childcare, the need to attend multiple medical appointments). Participants completed an online battery of self-report questionnaires which included four open-ended questions about the content of RT. Although much of the abovementioned literature has focused on RT that is negative in content, we opted to examine RT irrespective of its emotional valence, in line with theoretical accounts (e.g., Watkins, 2008). Participants’ responses to these open-ended questions about RT content were the focus of this paper.

## Materials and Methods

### Design

This qualitative study involved an online survey of mothers in the first year after the birth of their first child.

### Participants and Recruitment

A convenience sample of postnatal women (*N* = 236) was recruited via online channels including Facebook and Twitter, university mailing lists, social media groups for pregnant women/new mothers and websites relevant to expectant and new parents^1^. Study advertisements were also posted on university noticeboards on campus and surrounding areas. Potential participants responded to advertisements asking women whose first child was born within the last 12 months to participate in an online survey about their experiences of being a new mother. To be eligible to participate, respondents needed to be fluent in English and willing to provide consent. We did not impose any restrictions on participants’ geographical location, nor did we ask participants to indicate their country of residence. Participants were not required to be experiencing depression, anxiety or high levels of RT. Interested participants clicked on the link to the online survey, provided electronic informed consent and then completed the questionnaire battery. The survey was open between February and June 2017. The consent form and measures were administered via an online survey platform (Qualtrics, 2017; Provo, UT, United States).

A total of 251 new mothers commenced the survey. Fourteen of these women provided responses to some or all of the demographic questionnaires but did not commence the open-ended questions or any of the questionnaires^2^. One participant completed the open-ended questions, but her baby was outside the specified age range (i.e., was older than 12 months). The final sub-sample of new mothers included 236 participants who completed the open-ended questionnaires.

Participants were provided with the opportunity to enter a draw to win one of five $AUD50 gift cards (or an Amazon voucher for participants living outside Australia) for their participation. All procedures received ethical approval from UNSW Sydney Human Research Ethics Advisory Panel C (HREAP – Behavioural Sciences; application file number 2765). At the completion of the survey, all participants were provided with written information to debrief them about the aims of the study and were given contact details for relevant mental health support services and resources.

### Measures

#### Demographics and Personal Background Questionnaire

Participants provided information about age, ethnicity, highest education level attained, employment status and relationship status. To gauge perceived social support, participants were asked: *How many people are you able to rely on for support? This may be for practical help, emotional help, financial help, or assistance in helping you know or do things*

Participants also indicated whether they had experienced depressed mood (i.e*., felt depressed or down, or felt sad, empty, or hopeless, most of the day, nearly every day*) or anhedonia (i.e., *much less interested in most things or much less able to enjoy the things you used to enjoy, most of the time*) for a period of two or more weeks at any time in the past month, past year, or prior to the past year. Participants were only classified as having had a (probable) previous episode of depression if they indicated that the period of depressed mood and anhedonia fell within the same timeframe (i.e., in the previous month, previous year, prior to the past year).

Participants also indicated whether they had their child in the past 12 months and provided the age of their child. Where relevant, participants also indicated whether they had experienced any complications with falling pregnant or carrying their baby to term and/or with labor/childbirth, or whether their child had experienced any problems in the neonatal period.

We also administered the following battery of self-report questionnaires in order to characterize the sample in terms of their symptoms of psychopathology and tendency to engage in RT:

#### Depression Anxiety Stress Scales – 21-Item Version

DASS-21 was administered to index symptoms of depression, anxiety and stress in the past week (Lovibond and Lovibond, 1995). The DASS-21 subscales have excellent internal consistency (depression: α = 0.94; anxiety: α = 0.87; stress: α = 0.91; Antony et al., 1998). In the current sample, α = 0.87, 0.76, and 0.84, for depression, anxiety and stress, respectively. The DASS-21 has been widely used in perinatal samples (e.g., Miller et al., 2006), with good levels of internal consistency reported for each subscale. Cut-off scores for moderate levels of symptoms on the depression, anxiety and stress subscales are 14, 10, and 19, respectively (Lovibond and Lovibond, 1995).

#### Edinburgh Postnatal Depression Scale

EPDS; Cox et al. (1987) was included to specifically measure symptoms of perinatal depression. Scores range from 0 to 30. The EPDS was developed to screen for perinatal depression and has been validated in pregnant women, and widely used in the perinatal literature. Owing to the online format of our study and in order to meet the requirements of our ethics committee we removed the item asking about suicidality. As a result of this modification, EPDS scores in the current study could range from 0 to 27. Even with this modification, the EPDS possessed good internal consistency in the current sample (α = 0.87).

#### Repetitive Thinking Questionnaire – 10-Item Version

RTQ-10; McEvoy et al. (2010, 2014) is a transdiagnostic measure of perseverative thinking that was developed based on the items of existing measures of rumination, worry, and post-event processing. We modified the instructions of the RTQ-10 to direct participants to respond to the items ‘*in relation to thinking about your day-to-day experience of being a new mother and thinking about your baby.’* The RTQ-10 possesses excellent internal consistency (α = 0.89); in the current sample, α = 0.95.

#### Ruminative Response Scale (RRS) of the Response Styles Questionnaire

RSQ; Nolen-Hoeksema and Morrow (1991) is a 22-item instrument that assesses participants’ tendency to engage in depressive rumination – i.e., engage in a range of ruminative responses when feeling ‘sad, down, or depressed.’ The RRS is the gold standard instrument used to measure rumination in the depression literature. It possesses excellent internal consistency (α = 0.90, Treynor et al., 2003); α = 0.95 in the current sample.

#### Maternal Infant Responsiveness Scale

MIRI; Amankwaa and Pickler (2006) is a 22-item questionnaire which indexes maternal responsiveness to infant cues. For example, the items assess a mother’s feelings and beliefs about how she responds when caring for and spending time with her baby. The MIRI has good internal consistency (α = 0.87; Amankwaa et al., 2007). In the current sample, α = 0.83.

#### Open-Ended Questions – Content of RT Related to Motherhood

In order to examine the content of RT, participants were provided with a series of prompts and were asked to indicate (i.e., yes/no) whether they had experienced thoughts related to each one. If they indicated that they had, participants then had the opportunity to describe the thoughts in detail and/or give an example. Specifically, participants were given the following instructions:

*When thinking about your experiences of becoming or being a mother, there might be some things that you notice your mind going back to over and over again. Please indicate whether you have experienced any of the following types of thoughts when thinking about your baby and experience of motherhood.* The five prompts (based on Beck, 1992, 2002) included incongruity between the expectations and reality of pregnancy/motherhood, not being able to function as prior to becoming pregnant or having a baby, feelings of loss, feelings associated with making improvements, and an ‘other’ category. The rationale for using the first four prompts was to capture material that had previously been identified in the literature as commonly reported by new mothers (Beck, 1992, 2002), and to provide a starting point for participants to report their RT. We also included the ‘other’ category to give participants the opportunity to report the content of any RT they had experienced that was outside these areas.

### Procedure

The online survey took approximately 30–45 min to complete. First, participants provided demographic and background details, including information on current or previous depressive episodes. Next they provided information on the content of RT, and completed a series of validated self-report questionnaires which indexed levels of psychopathology symptoms (e.g., depression, anxiety), levels of RT, and mothers’ responsiveness to her infant.

### Ethical Approval

All procedures received ethical approval from UNSW Sydney Human Research Ethics Advisory Panel C (HREAP – Behavioural Sciences; application file number 2765). Given the online format of the study, participants provided informed consent by ticking a box.

### Data Analysis

Participant characteristics were analyzed using a quantitative approach to generate descriptive data for demographic details and standardized measures, conducted in SPSS. For the short answer component, thematic analysis was selected to analyze this data, due to its flexibility and rigor (Aronson, 1995; Fereday and Muir-Cochrane, 2006), which is well-suited to the current study because it allows for the identification, interpretation and reporting of patterns within the data (Tuckett, 2005). In line with the importance placed on reflexivity as part of this analytic approach (Braun and Clarke, 2019), we consider the perspectives and experiences we have brought to this project. We are five female clinician-scientists, with backgrounds in clinical psychology and mental health, and diverse research interests within these areas spanning experimental psychopathology to applied public health approaches. Each of us has knowledge and experience in research of repetitive negative thinking and how this relates to emotional disorder, albeit with differing levels of experience, spanning early (MB), mid (JN, AW-S), and senior-career academics and clinical psychologists (CH, MM). Our motivation in undertaking this research is to better understand the role of RT in the perinatal period - with a view to informing research and ultimately clinical practice, as a way to reduce the well-documented distress and anxiety often experienced by new mothers.

An inductive approach was adopted for this study, which involves a process of coding data without trying to fit it into a preexisting coding frame, theory or framework. However, this occurred within the broader context of a pragmatist perspective because our interests lie in the subsequent application of this work (Long, 2013), as informed by clinical psychology. Iterative, recursive coding process was used to code the free-text data. Initial familiarization of the data was achieved by reading through the responses multiple times and deeply engaging with the data, both individually and then through discussion. Initial coding was conducted independently by the two coders (JN, AW-S) on the entire dataset, using an inductive, data driven approach in order to generate an initial first-stage coding framework (Braune and Clarke, 2006; Neale, 2016; Braun and Clarke, 2019). The initial codes were developed by the two coders, and were then discussed and refined between the coders until there was agreement. In the second stage of coding, both coders then independently recoded a subset of the data to test the validity and appropriateness of the themes identified in this coding framework. Discrepancies in data categorization were resolved through discussion in order to create detailed code descriptions that could be applied consistently to the free-text responses. Attention was paid to unexpected themes or differences in responses between participants (Lather, 2012). This final coding framework was then applied to the dataset. Analysis involved summarizing the content within each coding theme, developing a description of each theme, and finally relating the key themes of the experience reported by participants both to other themes, and to the relevant literature (Braune and Clarke, 2006; Neale, 2016; Braun and Clarke, 2019).

## Results

### Participant Characteristics

A total of 236 new mothers completed the open-ended questions (see Table 1 for participant characteristics for both sub-samples). Whilst we do not discuss the self-report measures in relation to our thematic analysis, we have opted to report them in order to characterize the sample. DASS-21 total scores ranged between 2 and 92, with 11.54% of participants reporting depression, 18.72% anxiety, and 16.95% stress symptoms at a moderate level or above. Scores on the EPDS ranged from 0 to 22.

**TABLE 1:** Participant characteristics (*N* = 236).

**Age**	***M* = 32.05 *(SD* = 4.48, Range = 21–44)**
**Ethnicity**	
*% Caucasian*	70.2
*% European*	9.8
*% Asian*	8.9
*% Aboriginal Torres Strait Islander*	0.9
*% African*	0.4
*% Pacific Islander*	0.4
*% Other*	9.4
**Education (highest level)**	
*% Postgraduate*	38.5
*% Undergraduate*	35.9
*% Tertiary qualification (diploma, TAFE)*	18.4
*% High school*	6.4
*% Other*	0.9
**Current employment status**	
*% Maternity leave*	59.4
*% Employed full-time*	12.4
*% Unemployed*	12.0
*% Employed part-time*	9.0
*% Full-time student*	2.6
*% Other*	4.7
**Relationship status**	
*% Married*	76.1
*% Partnered*	17.9
*% Cohabiting*	3.4
*% Single*	1.7
*% Divorced*	0.9

**Perceived social support**	***M* = 18.12 *(SD* = 29.18)**

**Depressed mood and anhedonia for two or more weeks**	
*% Past month*	1.27
*% Past year (i.e., prior to past month)*	5.93
*% Prior to past year*	8.47

**Age of child (months)**	***M* = 5.93 *(SD 3.07*, Range = 1–12)**

**Complications**	
*% Complications with pregnancy (e.g.*,	19.7
*gestational diabetes)*	
*% Complications for mother or baby with labor/birth (e.g., hemorrhage in childbirth)*	13.9
*% Postnatal or neonatal difficulties (e.g., baby had meningitis)*	11.1
EPDS	*M* = 7.06 *(SD* = 4.76)
DASS-21 depression	*M* = 6.48 *(SD* = 6.70)
DASS-21 anxiety	*M* = 5.11 (*SD* = 5.82)
DASS-21 stress	*M* = 12.85 (*SD* = 7.43)
RTQ-10	*M* = 21.37 (*SD* = 10.54)
RRS	*M* = 38.36 (*SD* = 13.25)
MIRI	*M* = 99.02 *(SD* = 7.9)

*EPDS, Edinburgh Postnatal Depression Scale; DASS-21 depression, Depression Anxiety Stress Scales – depression subscale; DASS-21 anxiety, Depression Anxiety Stress Scales – anxiety subscale; DASS-21 stress, Depression Anxiety Stress Scales – stress subscale; RTQ-10, Repetitive Thinking Questionnaire – 10 item version; RRS, Ruminative Response Scale of the Response Style Questionnaire; MIRI, Maternal Infant Responsiveness Scale. Owing to missing data, *n*s vary across measures; range from *n* = 202–236. Where relevant, percentages refer to the proportion of participants who responded to that item.*

Four participants responded ‘no’ to all of the prompts about possible themes of RT, and did not provide any responses to the open-ended question asking participants to describe RT content. The remainder of the sample provided responses to indicate RT content in response to at least one of the prompts. Notably, a number of participants responded ‘no’ to a prompt, but nonetheless provided an open-ended response to the prompt in which they described RT content, perhaps suggesting that they were unable to recognize that they had engaged in RT, or an inclination to not label it as such.

### Thematic Analysis Results

There was conceptual overlap between the prompts. Therefore, we collapsed the responses across the prompts used, and performed a thematic analysis on the entire set of responses to the open-ended questions, which generated 369 unique responses for coding. The thematic analysis generated the following five broad themes: (i) range of emotions experienced during (i.e., ‘emotional spectrum’ of) motherhood, (ii) discrepancy between expectations and reality, (iii) changes in the self and relationships, (iv) feeling trapped, and (v) preparedness to seek support. Several sub-themes were identified within each broad category, and all are listed in Table 2, with illustrative quotes listed for each sub-theme, together with the proportion of responses that fit within that theme. We present the themes in the order in which they appeared to the authors to be of importance to new mothers.

**TABLE 2:** Themes, subthemes, and illustrative quotes for each theme that emerged in the thematic analysis (percentages in parentheses).

**Theme**	**Sub theme**	**Examples**
**1. Emotional spectrum of motherhood (30%)**		
	1.1 Strong emotions (23%)	Now that my baby is here I am lonelier than I’ve ever been even though I am with her all the time …. I was still surprised at the intensity of the loneliness and boredom at times, the monotony and the massively fluctuating emotions (I’m normally very level). My emotions are more heightened and feelings of being overwhelmed and ‘trapped ‘ were unexpected. Feel gloomy all the time and whilst I feel alone it’s to hard to make effort to correct the situation as I feel like I’m boring and have nothing to talk about
	1.2 Absence of expected emotions (4%)	Falling in love with or feeling affection for your baby can take weeks. I thought it would be more intense on both sides. I guessed happy moments would be like a drug filled high and down moments would be like being at war. Overall it was great, but not the high I thought when people told me I couldn’t imagine it….turns out I could imagine, I just believed them and thought it would be more than this. Once I had them I didn’t really feel the magic that I was hoping for a while didn’t know how to bond with my tiny treasures. I knew motherhood would be hard but I thought I’d like it more than I do. Being a mother is not a natural thing for me, and I am less content with it then I feel I should be
	1.3 Unexpected emotions (3%)	I didn’t expect to experience feelings of sadness, loneliness and hopelessness in the early stages after giving birth. Being a mother brings me more joy and happiness than I expected
**2. Discrepancy between expectations and reality (40%)**		
	2.1 Housework (4%)	Managing household duties - unable to get anything done around the house with babies (lack of) sleep and constant need to be held I didn’t expect my baby to be so clingy and demanding to be carried all the time so I can’t get much done around the house I struggle to achieve everything I want in the day. Between fatigue and trying to multitask with her, family, husband that works long hours, returning to work (in a new job) I feel some days I am barely functioning.
	2.2 Career/paid work (4%)	Difficulty adjusting to position of authority and sense of purpose in working life to Day to day inanity of life at home with a baby and concerns about competence to return to work after extended absence. Concerns of leaving baby with others, entering daycare on my return to work, anxiety around adjusting to being away from baby to go back to work I feel that society views it as unacceptable for a mother not to go back to work after having a child. It’s not “are you,” it’s “when are you.” And although from a feminist POV that’s all very nice, it makes the choice to dedicate the next few years in particular to raising a functioning member of society seem unacceptable. I feel I have to justify it/not show any signs of financial or mental weakness as I don’t want the, “well if you had gone to work,” crap.
	2.3 Lack of achievements during leave (13%)	I would like to work more or do more study, but can’t find the energy or desire to actually do it. I thought I would have time on mat leave to do other things, little projects but nope.
	2.4 Breastfeeding (5%)	Breastfeeding in reality is difficult, hard work There was not an immediate bond with my baby and breastfeeding wasn’t the beautiful natural bonding experience it’s perceived to be. It’s hard and painful. And the connection with my baby was not immediate. Breastfeeding is the most complicated difficult thing of motherhood - the pressure to do it, the inclusiveness while you feed and the isolation and lack of support when it doesn’t work. I experienced grief and loss in relation to not being able to breastfeed…. I felt that by not breast feeding I was letting my baby down and was worried about being judged and misunderstood by friends / the broader community, particularly as all my friends have breastfed their babies.
	2.5 Doubt about capacity as a mother (9%)	I am not always acting like the mother I wanted to be- not mindful enough, not creative enough, not always in tune with my baby, not creating an environment that is stimulating and supports independence etc. I feel like a terrible mother who isn’t doing the best for her baby as I don’t know what I’m supposed to be doing and whether it’s right or not. I am not the mother I expected myself to be.
	2.6 Baby-related expectations (5%)	And she is very different too. She is very confident and loud I thought my baby would be easy to care for, that he would sleep, that I’d get housework done, that is have a short period of leave and return to my pre-baby life easily …. I didn’t expect my baby to be so clingy and demanding to be carried all the time so I can’t get much done around the house
**3. Changes in the self and relationships (40%)**		
	3.1 Physical (4%)	Physical changes to body post labour, slow healing, aches and pains. My body and life will never be the same.
	3.2 Cognitive Changes (including sleep/fatigue) (16%)	Nothing prepared me for the isolation, health challenges after particularly weight related, coping with being needed constantly and the constant fatigue I didn’t expect to be quite this sleep deprived. I am tired all the time & complain about how tired I am, what a bad night I had, how unhelpful my husband is, how baby won’t stick to routine, how I can’t go out, how I’m stuck at home…
	3.3 Changes in Identity (11%)	Ihave lost myself since becoming a mother… I don’t know where I belong in the world. No one cares about me or my baby anymore I know I am a mother, but I sometimes feel disconnected from seeing myself that way. I’m still adjusting to how being a mother impacts who I am to everyone else, and how everyone else sees me. Am I me with a baby, or am I just me? …. my identity has completely changed but that’s not a negative thing to me, not a loss as such, a change. I feel more like ‘old’ me is here, just on a top shelf like a winter coat in a warm climate. I do sometimes feel disheartened about the feeling of turning into a beige middle-aged mum. At times I’ve felt I’m not valued as a person beyond being “the host” to my child and then “the chauffeur” …. I’m kind of invisible now professionally and out and about..at work people have no interest in giving me opportunities to grow. Out and about my child has become my identity. im still not happy with this. I wish people would recognise that I’m an accomplished person as well as being a mum.
	3.4 Changes in Relationships (9%)	**Partner** I was also not prepared for the massive change that would occur in the relationship between my husband and I, I feel like we have only just found our “happy place” as a couple again 11 months later, but at the same time the relationship is different now and will never be how it used to be. This is not necessarily bad just different. The fact that my partner and I don’t even know how to be friends ATM because we’re so tired. The fact that his non baby friends have no idea what it’s like and made no effort towards me post baby. But continued to treat him like his life hadn’t changed. He doesn’t even seem to care how exhausted I am as he works. He doesn’t see the extra work I do. His expectations in our relationship is crazy so I struggle to get the time I need for myself. The only struggle I have had is keeping the spark alive with my partner once a baby arrives. I love motherhood and have had no regrets. But wish I felt sexy again to my partner I guess that will cone with time. **Friendships** …. isolation from non-parent friends, loneliness… Adjusting to friends expectations who do not have children and understand the demands on a new lifestyle Guilt- guilty that I had my beautiful baby while some close friends had serious health issues with their babies
**4. Feeling trapped (22%)**		
	4.1 All-consuming nature of motherhood (12%)	The sheer demands of looking after a baby and post natal recovery, especially in the first few weeks, can be overwhelming. The 24 hour nature of being a mother was so much harder than I expected. I hadn’t realised how much of me is required… 100% of me 100% of the time. I was not prepared for that. Feelings of being overwhelmed and trapped were unexpected.
	4.2 Inability for spontaneity (5%)	….[I] wasn’t quite prepared to never have a break. I’ve lost my freedom Life with my baby is different to what I expected- it is much harder and complex. I love it but it has been a huge adjustment. I have found the loss of spontaneity very difficult, everything has to be planned out in advance now. I can’t do what I usually do, ie. go out, see my girlfriends, exercise, shop etc. I have no control over my time, body, energy, sleep or whatever I usually do to relax.
	4.3 Mourning the loss of ‘old life’ (4%)	My lifestyle, my freedom and my energy are gone. I wonder if I will have the career I dreamed of because that’s paused at the moment. Will I ever have my old life back? Grieved my old life but now love new life Most of the time it isn’t a negative feeling of my life not being my own anymore, bit there are definite pangs of jealousy when I see other people going out or making plans on a whim
	4.4 Feeling trapped financially (1%)	With all this I am in a hugely vulnerable position emotionally as well as financially. And [because] I don’t feel like I have a stable ground at the moment my personal finances stress me out ie if we were to split I would survive but I’d also be struggling. Only in that as I no longer work I do not earn my own money and have to rely on my husband, I find this quite difficult, especially being an older mum
**5. Preparedness to seek support (12%)**		
	5.1 Active help seeking (8%)	Seeking advice from health professionals and other mums Constantly reading on what I can do to become a better mother: helping with development, foods to feed, sleep patterns etc
	5.2 Avoiding help seeking (3%)	I feel like people will think I am incompetent if I ask for help. That asking for help is a sign of weakness or that I am not managing What daily pattern should i be setting for my baby? I need to know/read about whether it’s ok to let him fall asleep on me. I can’t look like i don’t know what to do for my baby in front of others. I will look as though I’m incompetent if my baby screams in front of other people. I need to learn as much as i can/time things properly so that doesn’t happen. Sleep- not wanting to admit that I can’t handle it. In initial stages not wanting to ask for help. Should be able to do it all!!! I don’t know how to ask for help
	5.3 Lack of support (1%)	I have nobody to ask for help except my partner

#### Theme 1: Emotional Spectrum of Motherhood

This theme refers to RT about the experience of unexpected emotions, and the intensity of these emotions, as well as the absence of expected emotional responses to motherhood. Thirty percent of responses were coded within this theme.

##### Strong emotions

The most commonly reported experience across all responses (23% of all responses) was the fact that new mothers described their emotions as being intense, quickly changeable, heightened, unexpected, and at times difficult to control. There was a vast range of intense emotions, including anxiety, anger, loneliness, grief, loss and trauma. A majority of new mothers reported RT about feeling ‘*completely overwhelmed*’ with the challenges of motherhood, describing RT about feeling anxious, fearful and worried about being judged by others, doubt about their own abilities as a parent, and worrying a lot about the future. There was also a common theme of feeling isolated, and a sense of loneliness. Some reported depressive feelings such as helplessness and sadness, and feeling *‘gloomy’*, *‘miserable,’* and like a *‘failure.’* Some described thinking about the negative toll that motherhood had taken on their overall emotional health and ability to cope. Some struggled with feelings of trauma related to the birth, and the grief and loss associated with the transition to motherhood. Others reporting thinking about anger, frustration, and irritability, and that they *‘snap’* more easily. A minority described not *‘being prepared’* for the intensity of the emotions and the effect of having a new baby on their emotional health.

##### Absence of expected emotions

Some (4%) of responses reported RT about the absence of emotions that they expected to feel, or feeling less intense emotions than they expected. Some participants reported RT about the absence of overwhelming love and a lack of immediate connection or bond with the baby that they had expected to feel, which was not as *‘natural’* as they expected. For example, one mother reported ‘*falling in love with, or feeling affection for your baby can take weeks.’* In contrast, other participants described feeling more relaxed, and less anxious, depressed and overwhelmed than they expected to feel. Finally, others reported engaging in RT about the opposite; that they had experienced less intense emotions, both negative and positive, than they had expected to feel. For example, one mother reported *‘I thought it would be more intense on both sides. I guessed happy moments would be like a drug filled high and down moments would be like being at war.’*

##### Unexpected emotions

A minority of new mothers reported unexpected emotions, including struggling with unexpected feelings of depression, hopelessness and loneliness, fear, and the tendency to feel stressed about *‘every little thing.’* Others described feeling more positive emotions, that they were grateful for their experience, that motherhood was more rewarding and *‘lovelier’* than they anticipated, and that they felt more joy and love than they had expected they would. Of all the responses provided, only 3% reported experiencing unexpected emotions.

#### Theme 2: Discrepancy Between Expectations and Reality

This theme refers to the areas in which there was a discrepancy between what new mothers expected from motherhood, and the reality of their experience. Six sub-themes were identified, which included the following broad domains: (i) housework, (ii) career/paid work, (iii) lack of achievement during leave, (iv) breastfeeding, (v) doubt about one’s capacity as a mother, and (vi) baby-related expectations. Forty percent of responses were coded within this theme.

##### Housework/chores

Although some participants reported RT about the idea that motherhood was easier than expected, the majority noted RT about finding it significantly harder than they expected and some (4%) thought frequently about how they were not meeting their own expectations in terms of household duties, chores, and everyday tasks.

##### Career/paid work

A small proportion of responses (4%) described a discrepancy between their expectations in terms of motherhood and paid work. Some new mothers described a strong desire to return to work, which they felt guilty about, in order to have a break from their baby. Some reported worrying about the negative impact of motherhood on their career and job prospects, as well as worry about their abilities at work. Fears of being perceived as incompetent, invisible, and less accomplished or capable than previously were significant sources of RT. A minority expressed concern about the negative impact of returning to work on the baby, including how the baby would cope being placed in daycare, and being separated. Lack of flexibility in work roles, and gendered roles with regards to returning to work was also mentioned as a theme of RT.

##### Lack of achievement during leave

Some (13%) participants reported RT about how they had not achieved as much during their maternity leave as they had expected in terms of hobbies, exercise, household improvements and other activities. For example, one mother reported: ‘*I thought I’d have more time to do extra things like DIY projects, starting a business, meeting up with friends etc. there’s less down time with baby than I expected.’*

##### Breastfeeding

Some participants (5%) also described RT about the unexpected challenge of and difficulties with breastfeeding, described as ‘*harder than I expected,’* ‘*painful,’* and ‘*difficult.’* Some reported RT about grief and having a sense of loss related to not being able to breastfeed, which was associated with feelings of failing their baby.

##### Doubt about capacity as a mother

Many participants noted RT about not feeling good enough, ways in which they had failed their baby, and had failed to meet their own expectations of how they would be as a mother. Nine percent of all responses described doubt in their perceived capacity as a mother. These perceived failures were across a range of areas, such as not being present enough, playful or fun enough, not available enough, or simply *‘not enough’* for their babies. Some described RT about the guilt associated with these concerns, and general uncertainty about whether or not they were doing the right thing or making the right choices. For example, one mother wrote *‘not knowing what is going on in my baby’s head makes me wonder if I’m doing the right thing or doing enough.’*

##### Baby-related expectations

Several new mothers (5%) described RT about their baby and how their baby was different to what they expected; specifically, crying more and sleeping less than expected, being more demanding or experiencing unexpected illnesses, reflux or other issues which they found anxiety-provoking. Comparisons with other babies and how *‘easier and calmer’* they appeared were noted as content of RT by participants.

#### Theme 3: Changes in the Self and Relationships

A major theme (40%) that was identified in the analysis was RT about the profound changes that new mothers reported experiencing in the postnatal period. There were six subthemes identified from the analysis which identified different domains of change: (i) physical, (ii) cognitive, (iii) identity, (iv) invisibility, (v) identity, (vi) relationships, and (vii) lifestyle.

##### Physical changes

Some (4% of responses) described RT about the physical changes they had experienced following labor, including pain and the fact that their recovery following childbirth took far longer than they expected. Others reported RT about losing control over their body, for example, one mother wrote *‘my body and life will never be the same.’* Others reported RT about weight gain and the loss of positive body image. RT about physical pain and discomfort was also common (e.g., *‘my body aches all the time’*).

##### Cognitive changes (including sleep/fatigue)

Participants described RT about cognitive changes including constant fatigue, exhaustion and the negative effects of sleep deprivation. Sixteen percent of responses described RT about how fatigue affected *‘everything’* including their concentration, ability to focus, memory, distractibility, and ability to work and function as well as they had before having a baby (e.g., ‘*I have found motherhood exhausting beyond expectations’*).

##### Changes in identity

A majority of participants reported RT about the loss of their sense of self. Others reported RT about how their identity had changed and they had lost their ‘old self’, or that they were unsure and figuring out who they were now that they had become a mother. In addition to the RT that participants reported about their own identity, several reported that they felt completely invisible and that others saw them as a *‘milk machine’* or a ‘*host.’* As a part of this sub-theme, several participants reported that since having a baby, they were now more likely to be overlooked for opportunities at work. Eleven percent of responses were coded with this theme.

##### Changes in relationships

New mothers described thinking a lot about unexpected changes to their relationships (9% of all responses described these changes), including those with their partners and friends, and to a lesser extent, other family members. Many participants reported RT about the changes in their relationship with their partner, about the fact that the relationship is ‘*different now’* and that it *‘will never be how it used to be.’* Some noted the same themes of RT with respect to their friendships and described this as distressing. Notably, others reported a degree of acceptance around the changes in their relationships. Others reported RT about feeling isolated from their friends and struggling to adjust to the different expectations of their ‘non-mum’ friends.

#### Theme 4: Feeling Trapped

The fourth theme that was identified in the analysis was RT about feeling trapped in the role of new a mother, reported in 22% of responses. The sub-themes predominantly captured the perceived relentlessness of motherhood and how participants felt that they were no longer able to decide how to spend their time, and in some cases, their money. Subthemes were: (i) the all-consuming nature of motherhood, (ii) inability for spontaneity, (iii) mourning the loss of their old life, and (iv) being trapped financially.

##### All-consuming nature of motherhood

Many participants (12%) reported RT about the 24/7, all-consuming nature of motherhood, including the challenge of coping with the constant demands of this role and schedules throughout the day and night. They described thinking about being consumed by the demands of caring for a baby, being with the baby all of the time and the ‘*lack of respite.’* RT was also reported about participants’ inability to find time to themselves, and focusing their entire life around the baby, and what that meant for their life, future, and identity.

##### Inability for spontaneity

Some participants reported RT about having a lack of freedom and spontaneity, and their inability to *‘escape’* from their baby. The feeling of being stuck at home, without being able to go out on a whim, trapped in their baby’s routine and needs was a common theme. While some expressed a sense of acceptance of the life adjustments, others had a stronger emotional response including feeling overwhelmed and resentful. Five percent of responses mentioned the inability for spontaneity as the content of their RT.

##### Mourning the loss of ‘old life’

Some participants (4%) described RT with a sense of mourning the loss of their old life, their friendships, and their old lifestyle. New mothers reported a sense of missing their freedom and independence, and knowing that they can’t get their old life back (e.g., *‘My life is changing and will never be the same again’)*. For some, these thoughts were fleeting, but for others it was a longer lasting a sense of grief, intense loss, and mourning. The majority of new mothers expressed sadness and mourning about their changed lifestyle, activities and old life, including their inability to engage in social activities, physical activity/exercise, partying or socializing, relaxing/pleasurable activities, or to pursue other sources of interest or curiosity.

##### Feeling trapped financially

Although only mentioned by four participants (1%), these individuals reported RT about feeling trapped due to being in a vulnerable financial situation, and being reliant on their partner for money. They reported coming to the realization that if they were to separate from their partner they would be in a tenuous financial position. They reported a lack of independence in being reliant upon partner for money with consequences for their emotions *‘Knowing I don’t get a pay check makes me feel a little inferior at times’* and ‘*My personal finances stress me out, i.e., if we were to split.’*

#### Theme 5: Preparedness to Seek Support

The final theme identified in the analysis was RT about help-seeking (12% of responses), with participants divided in terms of whether they reported seeking or avoiding help, and their experiences of support as a new mother.

##### Active help seeking

Some women (8%) reported RT about actively seeking out help from other people including counseling or psychological support and seeking out and reading information (e.g., about how to improve their baby’s sleep) in order to learn.

##### Avoiding help seeking

Other new mothers (3%) reported RT about avoiding help-seeking or information-seeking due to fears of looking incompetent if they asked for help, as well concerns as to what this would say about them *‘Asking for help is a sign of weakness or that I’m not managing’* and *‘Asking for help meant I was a failure.’* Others expressed confusion and uncertainty about who and where to ask for help, as well as guilt about asking for help in the first place.

##### Lack of support

A few new mothers (1%) reported RT about the lack of support in their networks; these were often women from overseas who had little family, social and emotional support in Australia.

## Discussion

Despite a growing body of evidence that RT plays a role in psychological adjustment in the perinatal period, the themes that characterize RT (whether rumination or worry) in postnatal women have not been investigated. Our thematic analysis identified five broad themes, namely: the emotional spectrum of motherhood, discrepancy between expectations and the reality, changes in the self and relationships, sense of feeling trapped, and preparedness to seek support. Broadly, these themes convey an overall sense of a discrepancy between expectations and reality of motherhood for many participants, as well as adjustment to profound change across multiple domains (personal and professional), and in many cases, a significant sense of loss. A loss of identity and experiences of strong emotions were most commonly reported. We note, however, that not all RT was negative: within these themes, a proportion of participants described positive content (e.g., unexpected positive emotions and experiences of motherhood). These themes, along with the related sub-themes, provide key insights into the content of repetitive thinking experienced by a community sample of postnatal first-time mothers. It is important to acknowledge that while we were driven by the open-ended responses provided by participants, our backgrounds and experiences may have influenced the way that responses were categorized into themes. That is, we cannot rule out the possibility that our roles as clinician-scientists with backgrounds in psychology may have affected the questions we asked, as well as the way in which we categorized the participant responses into themes. In future work, it would be useful to involve people with different backgrounds, including professional (e.g., nursing, midwifery), and personal (e.g., lived experience of perinatal anxiety or depression) in the interpretation of the data.

To the extent that key theoretical models of RT (e.g., Watkins, 2008) and clinical interventions which target RT (e.g., behavioral activation; Martell et al., 2001) primarily focus on the process of RT rather than on its content, the absence of research on the content of RT in perinatal adjustment is perhaps not surprising. Further, it mirrors a similar lack of such work in the broader clinical literature. However, we see significant potential value in identifying RT themes. Although there will no doubt be variability in new mothers’ experiences and responses, the emergence of consistent themes of RT in our sample highlights important commonalities. For example, while effective psychological treatments for postnatal depression and anxiety are emerging in the literature (Sockol, 2015; Loughnan et al., 2019), our identification of commonly experienced themes of RT in first-time mothers has the potential to assist clinicians to refine and tailor these interventions in the future in order to effectively target RT within such protocols (e.g., by providing relevant, pertinent examples to illustrate RT).

Our findings complement those of previous work which has investigated the link between perinatal depression/anxiety and the content of thinking reported in the perinatal period, including maternal attitudes (Sockol et al., 2014) and rigid parenting-specific beliefs (Thomason et al., 2015). Indeed, the cognitions associated with beliefs about motherhood in these studies strongly resonate with the themes which emerged from our analysis. Similarly, O’Mahen et al. (2012) identified unmet high expectations as a theme of negative thoughts influencing depression in the perinatal period – for example, the discrepancy between women’s expectations of motherhood versus their actual experience. Our study adds to this work by demonstrating that these types of themes feature in the RT experienced by postnatal women, and highlights the value of examining the interplay of the content and process of cognition in the perinatal period to inform both prevention and treatment approaches. Specifically, by identifying cognitive content that is characteristic of perinatal RT, our findings offer clinicians (along with practitioners from other disciplines working with perinatal women – e.g., pediatricians) examples of patterns and content of thinking which can be screened (e.g., using self-report measures) in both expectant as well as new mothers, e.g., as part of routine antenatal/postnatal appointments. In turn, they can be targeted in preventive approaches and early interventions, with the goal of preventing long-term psychological difficulties in the perinatal window.

Whilst our sample was unselected and drawn from the community rather than a clinical population, the self-report measures indicated that 12, 19, and 17% of participants reported at least moderate levels of depression, anxiety and stress, respectively. Although the majority of our sample was therefore in the normal range, it is noteworthy that RT was common such that with the exception of only four respondents, some RT content was reported by all participants in the open-ended questions. This suggests that RT is common in first-time new mothers irrespective of the presence of psychological symptoms, and highlights the potential value in intervening to reduce RT and its associated distress in a period of heightened vulnerability for the development of psychopathology.

Many questions remain in this line of investigation. For example, the themes of RT in women who are pregnant with their first baby are yet to be examined. It is plausible that the themes of RT reported above may overlap with the future-focused worries of women who are expecting their first child; for example, worries about one’s capacity to cope. That said, it also seems likely that there will be differences in the content of RT in these two periods, owing to the distinct issues and specific challenges they each present. For example, in the antenatal period, an expectant mother may engage in RT about the possibility of miscarriage, and later, the upcoming birth experience. A longitudinal study that examines RT in a sample of pregnant women and again during the postnatal period would shed light on the extent to which the themes of RT shift during the perinatal period, or remain broadly consistent throughout. Relatedly, it would be interesting to investigate the content of RT in women who are pregnant with a subsequent child, and to compare that with the focus of RT in first-time mothers. Further, the extent to which the content of RT postpartum reflects (and is potentially an exacerbation of) longstanding beliefs about the self, and whether the content and focus of RT shifts (if at all) throughout the postnatal period, represent future areas of study. Finally, although we have focused on mothers in this paper, these questions await investigation in fathers, particularly in light of recent evidence of the prevalence of postnatal psychological difficulties in first-time fathers (e.g., Da Costa et al., 2019).

We note some limitations of the current study. The primary limitation is that whilst we sought to capture the content of RT by instructing participants to report thought content that they noticed ‘going back to over and over again,’ we cannot rule out the possibility some participants may have reported general thoughts about, and affective experiences associated with, new motherhood, rather than RT (i.e., thoughts that occurred repeatedly). The fact that a small proportion of participants indicated that they did not engage in RT, yet provided an open-ended response reporting RT content, is suggestive of this possibility. Future studies will be improved by checking participants’ understanding of the distinction between RT and general thoughts at the outset. Second, by providing participants with specific prompts to illustrate possible content of RT, we acknowledge the possibility that we may have inadvertently shaped the themes and content which emerged from our analysis. Moreover, the somewhat negative tone of these prompts may have reduced the likelihood of participants providing positive examples of RT themes. Future studies that employ a completely open-ended response approach will remove these concerns and confirm whether the same pattern of responses emerge. Third, participants were recruited via an online survey and were thus (intentionally) drawn from a community (rather than treatment-seeking) sample, and as such, varied in terms of their depression and anxiety severity. Although this variability is advantageous in terms of enhancing the representativeness and size of the sample, we do not know whether the same themes would emerge in a clinically depressed or anxious sample, or in a sample of healthy women. Fourth, we employed an online survey to gather open-ended responses to prompting questions. Whether similar findings would emerge in focus groups, or interviews to inquire about participants’ RT content is unknown. Fifth, without obtaining information about participants’ geographical location, we acknowledge that our data cannot speak to cultural differences that potentially shape women’s experiences in the perinatal period, and may emerge as themes in their RT. Finally, whilst the RTQ-10 and RRS have been widely used to index RT in a broad range of clinical and non-clinical populations, to our knowledge they have not been validated for use in perinatal samples. Validation studies, along with studies in which researchers develop instruments which specifically index RT in the perinatal period, will be important next steps in this line of work.

These limitations notwithstanding, to our knowledge this study is the first to explore the themes of RT in postnatal first-time mothers. The themes that emerged provide an important starting point to guide future studies of RT in postnatal anxiety and depression, and in turn, to inform the focus of interventions that seek to reduce RT in these conditions.

Despite evidence of the predictive and maintaining role of RT in depression and anxiety, the nature and role of this cognitive process in the context of perinatal psychological adjustment has only recently begun to receive research attention (DeJong et al., 2016). Noting the absence of research on the content of RT in the postnatal period, we explored the themes of RT in a sample of first-time mothers and identified five key themes. Together, the themes conveyed a sense of discrepancy between expectations and reality, and participants’ adjustment to profound change. Going forward, our identification of these themes has scope to assist clinicians to tailor interventions for postnatal psychological difficulties.

## Data Availability Statement

The datasets presented in this article are not readily available because of the need to preserve participants’ anonymity. Some quotes from the content of the open-ended questions are reported in the paper in order to illustrate the themes that were identified – however, the full original transcripts are not available to the public in order to retain participants’ privacy and anonymity. Requests to access the datasets should be directed to MM, m.moulds@unsw.edu.au.

## Ethics Statement

The studies involving human participants were reviewed and approved by UNSW Sydney Human Research Ethics Advisory Panel C (HREAP – Behavioural Sciences). Written informed consent was not provided because the study was completed online, thus participants were not able to provide written consent. However, all participants ticked a box to give their informed consent to take part. This consent procedure was approved by the ethics committee.

## Author Contributions

All authors conducted the study as part of a larger collaboration, and read and approved the final version. MM and MB designed the study. MB programmed the survey content. JN and AW-S coded responses to the open-ended items. JN, AW-S, MM, MB, and CH contributed to writing the manuscript.

## Conflict of Interest

The authors declare that the research was conducted in the absence of any commercial or financial relationships that could be construed as a potential conflict of interest.

## Footnotes

1 The survey also included additional self-report questionnaires that are peripheral to the aims of the study described in this paper. We report the data relevant to these measures in another paper, in which we adopt a quantitative approach.

2 There was no difference between the group of participants who completed the open-ended questions and those who did not, in terms of mothers’ age, baby’s age, social support, depression, anxiety, and stress (*p*s < 0.05).
